# Changes in environmental conditions regulate the biodiversity of planktonic microeukaryotes mediated by the dispersal-selection relationships in river: an example of the Beipan River, Guizhou, China

**DOI:** 10.3389/fmicb.2025.1649800

**Published:** 2025-08-19

**Authors:** Xiaohan Dong, Jiaxin Huang, Xinxin Zhou, Jiali Ran, Ziwei Wang, Zongqiang Qi, Yanjun Shen

**Affiliations:** ^1^Laboratory of Water Ecological Health and Environmental Safety, School of Life Sciences, Chongqing Normal University, Chongqing, China; ^2^Chongqing Key Laboratory of Conservation and Utilization of Freshwater Fishes, Chongqing, China; ^3^Animal Biology Key Laboratory of Chongqing Education Commission, Chongqing Normal University, Chongqing, China

**Keywords:** biodiversity, environmental DNA, phytoplankton, zooplankton, community assembly

## Abstract

River planktonic microeukaryotes (phytoplankton and zooplankton) underpin aquatic ecosystem function, yet how environmental change regulates their biodiversity via assembly mechanisms remains poorly understood. Using eDNA metabarcoding along China’s Beipan River, partitioned by a barrier dam into environmentally heterogeneous upstream and stable downstream regions, we assessed plankton diversity and the roles of dispersal and environmental selection. Phytoplankton exhibited higher alpha- and beta-diversity than zooplankton, attributed to stronger dispersal but weaker selection. Conversely, zooplankton showed higher gamma-diversity, likely due to broader niche breadths amplified by environmental heterogeneity. Upstream sites displayed significantly greater alpha-, beta-, and gamma-diversity for both groups, driven by higher environmental heterogeneity. Environmental selection dominated community assembly throughout the river, particularly influencing phytoplankton diversity. Dispersal contributed more to zooplankton gamma-diversity in the homogeneous downstream region. Biodiversity correlated strongly with environmental conditions, especially with COD and TOC levels in the variable upstream zone. Our findings demonstrate that environmental heterogeneity governs plankton biodiversity by regulating the dispersal-selection balance, providing new insights into assembly mechanisms and responses to global change in river ecosystems.

## 1 Introduction

River ecosystems have been shown to be of critical importance in sustaining biodiversity (Naka et al., 2022), supporting human livelihoods (Fu et al., 2023), and mitigating environmental change (Yue et al., 2025). The conservation and sustainable management of these species must be accorded urgent priority in view of the ongoing threats to their survival (Madhav et al., 2022). Planktonic microeukarytoes, encompassing phytoplankton and zooplankton, form the foundation of riverine ecosystems by driving energy transfer, nutrient cycling, and ecosystem stability (Zhang et al., 2023). Phytoplankton, as primary producers, fix carbon dioxide and generate oxygen through photosynthesis (Wang et al., 2024), while zooplankton mediate energy flow by consuming phytoplankton and transferring biomass to higher trophic levels (Peipoch and Ensign, 2022). These communities exhibit heightened sensitivity to environmental gradients, with anthropogenic disturbances such as nutrient pollution, dam construction, and hydrological alterations exerting substantial influence on their diversity and functional roles (Engel et al., 2019; Xu et al., 2020). Simplification in these communities has the potential to compromise the carbon sequestration, water purification, and fisheries productivity that is vital to the region (Goździejewska et al., 2024; Keck et al., 2020; Oldenburg et al., 2024). Future management strategies should prioritize the safeguarding of microeukaryotic biodiversity. Effective monitoring of phytoplankton-zooplankton dynamics will enhance predictive models of ecosystem responses to global change.

Planktonic microeukarytoes are complex assemblages whose composition and function are shaped by dynamic ecological processes (Barbour and Martiny, 2024). The field of community ecology has developed a theoretical framework proposing four fundamental processes governing biodiversity patterns: selection, dispersal, drift, and diversification (Vellend, 2010). Among these processes, selection (deterministic forces such as environmental filtering) and dispersal (the movement of organisms across space) play more pivotal roles in structuring microeukaryotic diversity due to the rapid generation times, high dispersal potential, and sensitivity to environmental gradients of microeukaryotes (Wang et al., 2023). Recent advancements in molecular ecology and theoretical frameworks have elucidated that selection and dispersal function in a synergistic and antagonistic manner, contingent on environmental context (Menéndez-Serra et al., 2023), spatial scale (Cagle et al., 2024), and microbial functional traits (Fan et al., 2024). While deterministic selection frequently predominates in environments abundant in resources, the phenomenon of dispersal limitation has been demonstrated to amplify stochasticity, thus giving rise to divergent communities even in circumstances where the environmental conditions are comparable (Brown et al., 2020). Conversely, high dispersal rates have been shown to homogenize communities without eliminating selective pressures, thereby underscoring the interdependence of these processes (Evans et al., 2017). The strength of selection and dispersal on community assembly varies with environmental heterogeneity (Huber et al., 2020), effective community size (Ron et al., 2018), and organism traits (Zhang G. et al., 2021). A comprehensive understanding of selection-dispersal interactions in the assembly of microeukaryotic communities within river ecosystems is imperative for effective prediction of community responses to global change, as well as the effective exploitation of microbiomes for societal benefit.

Conventionally, the monitoring of planktonic microeukaryotes has been based on net sampling and microscopic identification (Lin et al., 2024). However, standard mesh sizes in nets have been shown to result in biased sampling, favoring the representation of certain plankton size classes, thereby underrepresenting smaller or larger organisms (Giering et al., 2022). Furthermore, conventional sample volumes have been shown to underestimate species richness, resulting in the omission of rare or less abundant taxa (Cermeño et al., 2014). Presently, the molecular method is employed to characterize the diversity and community composition of microeukaryotes by sequencing specific genetic markers based on environmental DNA technologies (Sun et al., 2023). It facilitates detailed taxonomic resolution and insights into community assembly processes across spatial and temporal scales in freshwater systems (Zou et al., 2024).

This study investigates planktonic microeukaryotes, encompassing phytoplankton and zooplankton, in the Beipan River in Guizhou, China. The investigation employs environmental DNA sequencing as a methodological framework. It was hypothesized that the assembly of phytoplanktonic and zooplanktonic communities, in addition to their response to environmental changes, would be different. In order to reveal this issue, an exploration was first conducted into the differences in biodiversity between different planktonic types and their variations along the river. Subsequently, the repercussions of dispersal and selection on their assembly, in conjunction with the influences of environmental changes in the dispersal-selection interactions, were further investigated. The findings of this study have provided new insights that will facilitate enhanced resilience to the impacts of global change on river ecosystems.

## 2 Materials and methods

### 2.1 Study area and sample collection

The Beipan River has its origins in the Wumeng Mountains in Yunnan Province, China, and is a primary tributary of the Pearl River. The river’s total length is 449 km, with a total drop of 1,985 m. The watershed is predominantly characterized by canyon-like mountainous terrain, characterized by significant declines and substantial water flow, thus classifying it as one of the regions with the most abundant rainfall in Guizhou, China. To date, six cascade hydropower stations have been constructed along the main stream of the Beipan River. Among these, the Guangzhou (GZ) Hydropower Station is the most substantial.

**Figure 1:**
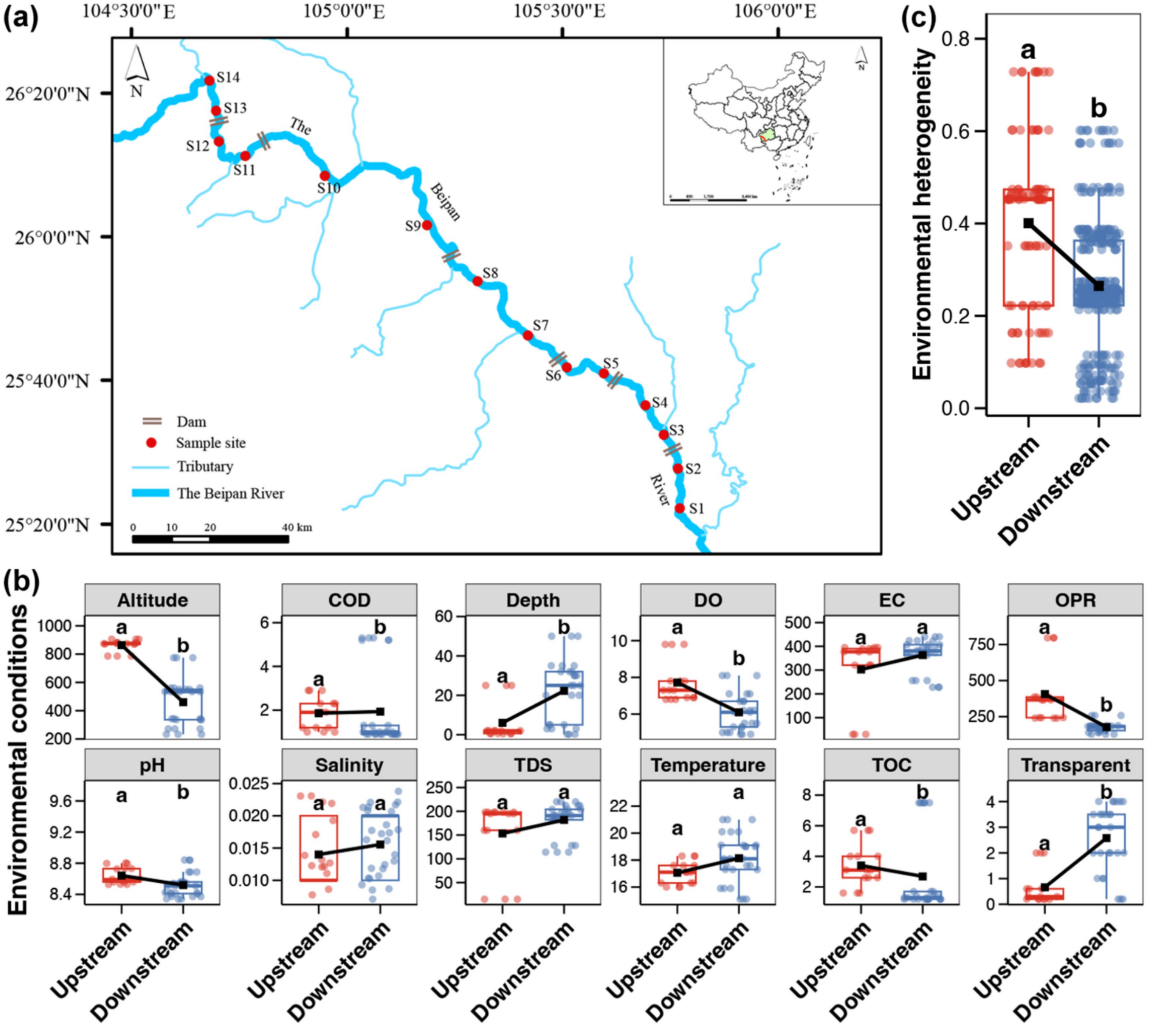
**(a)** Map of sampling sites. **(b)** Differences in the water environmental variables between the upstream and downstream of the Beipan River. A point represents a sample (*n* = 15 in upstream and *n* = 27 in downstream regions, respectively). **(c)** Differences in the environmental heterogeneity between the upstream and downstream of the Beipan River. A point represents a pair of samples (*n* = 105 in upstream and *n* = 351 in downstream regions, respectively). The black square represents the average value and the different lowercase letters above each box in the same subfigure represent significant differences between the upstream and downstream regions (Wilcoxon rank sum test, *p* < 0.05).

A total of 14 sites were selected for the collection of water samples along the Beipan River (see Figure 1a). The presence of the largest fully barrier-type dam, GZ Dam, between S8 and S9 has divided the Beipan River into two distinct regions, exhibiting significantly different flow velocities. Therefore, this study demarcates the research area into upstream and downstream sections based on the distinct aquatic habitat differences. At the upstream section (from S10 to S14), the river exhibits high flow velocity, thus establishing a habitat conducive to flowing water. Conversely, the flow rate of the downstream segment (from S1 to S9) is notably low, exhibiting characteristics reminiscent of a reservoir-like habitat. A total of three surface water samples from the relative depth strata were collected at each sampling site, one sample from the left, one from the center, and one from the right side of the sampling site. Approximately 2 L of surface water were sampled using a water sampler, and subsequently divided into two equal parts. One of the subjects was stored at a temperature of 4 °C for the purpose of measuring environmental variables. A further element of the procedure entailed the immediate filtration of water samples through a polycarbonate membrane with 0.22 μm pores in diameter (142 mm in diameter, Millipore, United States), utilizing a peristaltic pump. The filters were preserved in liquid nitrogen immediately, then transported to the laboratory and stored at −80 °C until DNA extraction.

### 2.2 Measurement of environmental variables

In the course of the sampling procedure, a series of environmental indicators were measured in parallel. The altitude of the sampling sites was observed by means of a GPS device. The water temperature and dissolved oxygen (DO) levels were measured *in situ* using an analytical instrument designated as the JPB-607A + analyzer. The pH, oxidation–reduction potential (ORP), and salinity were detected by means of the following instruments: a portable pH meter; a water quality tester (ORP-BL); and a salt concentration meter, respectively. The electrical conductivity (EC), total dissolved solids (TDS), chemical oxygen demand (COD) and total organic carbon (TOC) of water samples were monitored by means of the LS310 multi-parameter water quality analyzer (Shenzhen Linshang Technology Co., Ltd., Shenzhen, China).

### 2.3 DNA extraction, high-throughput sequencing, and data processing

The total DNA from each water sample was extracted from the filters using a PowerWater DNA isolation kit (QIAGEN, CA, United States) in accordance with the manufacturer’s instructions. Following the quality measurements by agarose gel electrophoresis (1% concentration) and NanoDrop ND-1000 Spectrophotometer (NanoDrop, United States), the qualified DNA were amplified by the primers 1389F (TCCCTGCCHTTTGTACACAC) and 1510R (CCTTCYGCAGGTTCACCTAC) to obtain the eukaryotic 18S rRNA V9 region (Amaral-Zettler et al., 2009). The detailed processes of PCR amplification and library construction were complied with and implemented by a previous study (Zhao et al., 2022). Subsequently, the libraries of all samples were subjected to sequencing using an Illumina NovaSeq 6,000 platform at Shanghai BIOZERON Co., Ltd. (Shanghai, China).

The raw sequences were subjected to a quality control process to obtain clean reads. This process involved the removal of reads with an average *Q* score of less than 20 and the deletion of reads that contained ambiguous bases or mismatches in the primers (Bokulich et al., 2013). Subsequently, the remaining high-quality reads were clustered into amplicon sequence variants (ASVs) using the DADA2 algorithm in QIIME2 software (Bokulich et al., 2018). In order to eliminate the effects of differences in sequencing depth, the read size of all samples was standardized to 500,000 reads. The SILVA 138.1 release (Yilmaz et al., 2014) was utilized as the reference database for taxonomic annotation of each ASV, and subsequently, ASVs belonging to phytoplankton and microzooplankton were assigned by a custom-written R script according to the method reported by Sun et al. (2023). Finally, to reduce the potential for sequencing error, ASVs that were only detected in one sample and had a read number lower than 10 were removed before further analyses.

### 2.4 Statistics analysis

All statistical analyses were conducted using R v 4.3.0 (R Core Team, 2022), and the results were visualized using the “ggplot2” package (Wickham, 2016). The Wilcoxon rank sum test was applied in order to evaluate the variations in environmental variables between the upstream and downstream regions. Furthermore, the environmental heterogeneity present in the upstream and downstream regions was calculated, respectively, based on the measured environmental variables according to the method reported by Doane et al. (2023). The “vegan” package (Dixon, 2003) was utilized to calculate the alpha- (Chao1 and Shannon), beta- (Bray–Curtis distance), and gamma-diversity of phytoplanktonic and zooplanktonic communities, respectively. The Wilcoxon rank sum test was utilized to estimate the differences in these biodiversity indices between phytoplankton and zooplankton. Concurrently, the Wilcoxon rank sum test was employed to evaluate the indices of a specific planktonic type, in addition to the relative abundances of predominant phytoplanktonic and zooplanktonic lineages between the upstream and downstream regions. Principal coordinates analysis (PCoA) (“ape” package, Paradis and Schliep, 2019) and adonis test (“vegan” package) based on the Bray-Curtis distance were further executed to assess the variations in the compositions of planktonic microeukarytoes between the upstream and downstream regions. Furthermore, a normalized core index (NorCI) (Zhang Q. et al., 2021) was calculated for each phytoplanktonic and zooplanktonic lineage to identify the core taxa in the Beipan River.

A neutral model was utilized to ascertain the significance of passive dispersal on community assembly by predicting the relationship between the frequency with which taxa occur in a set of local communities and their abundance across the wider metacommunity (Sloan et al., 2006). This analysis was conducted using non-linear least squares fitting in the “minpack.lm” package (Elzhov et al., 2010), and the m value in the model result is the estimated dispersal rate. The outlying mean index analysis was employed to estimate the effects of environmental selection on planktonic communities using the “ade4” package (Dray and Dufour, 2007). A high OMI value indicates that each phylotype has a narrow niche breadth, thus suggesting that each phylotype is subjected to higher environmental selection (Luan et al., 2020). The Wilcoxon rank sum test was employed to analyze the disparities in the dispersal and selection for phytoplankton and zooplankton. Linear regression was applied to evaluate the correlations between dispersal and selection with the biodiversity (alpha, beta, and gamma) of phytoplankton and zooplankton, respectively. The Rao quadratic entropy was employed to evaluate the contribution of alpha- and beta-diversity to the gamma-diversity of phytoplanktonic and zooplanktonic communities (Xiong et al., 2020). The Mantel test was finally employed to investigate the associations between alpha- and beta-diversity of planktonic microeukaryotes with environmental variables using the “linkET” package (Huang, 2021).

## 3 Results

### 3.1 Variations in environmental conditions

All measured environmental indicators are shown in the Supplementary Table S1. As demonstrated by the comparisons of environmental variables, the altitude, DO, OPR, pH and TOC content were found to be significantly higher in the water of the upstream location than in that of the downstream location (Wilcoxon rank sum test, *p* < 0.05, Figure 1b). In contrast, the downstream region of the Beipan River exhibited higher water depth and greater transparency in comparison to those in the upstream region (Wilcoxon rank sum test, *p* < 0.05, Figure 1b). Furthermore, a higher environmental heterogeneity was observed in the upstream region in comparison to the downstream region (Wilcoxon rank sum test, *p* < 0.05, Figure 1c), indicating stronger changes in environmental conditions in the upstream region.

### 3.2 Basic information of planktonic microeukarytoes

A total of 1,005,092 high-quality reads (average of 23,950 per sample) and 647,022 high-quality reads (average of 15,405 per sample) were obtained for the phytoplankton and zooplankton, respectively, in the Beipan River (Supplementary Table S2). The sequencing data have been deposited in the NCBI Sequence Read Archive (SRA) database under accession numbers SRR33930973-SRR33931014. The reads of phytoplankton were clustered into 8,933 ASVs with 100% annotation to a phylum, and 79.19% of these were assigned to a taxonomy at the genus level (Supplementary Figure S1a). In total, 75 families, 178 genera, and 59 species belonging to eight phytoplanktonic phyla were detected in the Beipan River (Supplementary Figure S1b). The rarefaction curve and species accumulation curve demonstrated that the sequencing depth and sample size could be representative of the intact phytoplanktonic communities in the Beipan River (Supplementary Figures S1c,d). For the purpose of this study, a total of 11,830 ASVs were obtained for the zooplankton sample, with 100% of these being annotated to a phylum and 89.94% being assigned to a taxonomy at the genus level (Supplementary Figure S2a). These zooplanktonic ASVs were annotated to 19 phyla, 272 families, 457 genera, and 198 species (Supplementary Figure S2b). The rarefaction curve and species accumulation curve further suggested that the sequencing depth and sample size could represent the whole zooplanktonic communities in the Beipan River (Supplementary Figures S2c,d).

### 3.3 Variations in biodiversity of planktonic microeukarytoes

**Figure 2:**
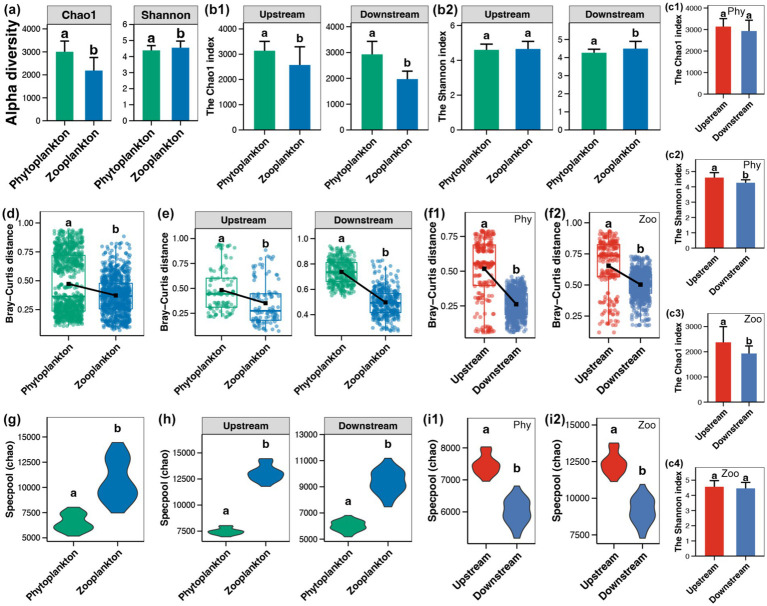
**(a)** Alpha-diversity between phytoplankton and zooplankton in the whole Beipan River. **(b)** Alpha-diversity (Chao1, **b1** and Shannon, **b2**) between phytoplankton and zooplankton in the upstream and downstream regions, respectively. **(c)** Alpha-diversity of phytoplankton **(c1,c2)** and zooplankton **(c3,c4)**, respectively, between the upstream and downstream regions. **(d)** Beta-diversity between phytoplankton and zooplankton in the whole Beipan River. **(e)** Beta-diversity between phytoplankton and zooplankton in the upstream and downstream regions, respectively. **(f)** Beta-diversity of phytoplankton **(f1)** and zooplankton **(f2)**, respectively, between the upstream and downstream regions. **(g)** Gamma-diversity between phytoplankton and zooplankton in the whole Beipan River. **(h)** Gamma-diversity between phytoplankton and zooplankton in the upstream and downstream regions, respectively. **(i)** Gamma-diversity of phytoplankton **(i1)** and zooplankton **(i2)**, respectively, between the upstream and downstream regions. Different lowercase letters in the same subfigure represent significant differences (Wilcoxon rank sum test, *p* < 0.05).

The initial study involved the calculation and comparison of two alpha-diversity indices of phytoplanktonic and zooplanktonic communities in the Beipan River. A significantly higher Chao1 index was observed in the phytoplankton sample, while the zooplankton communities exhibited a higher Shannon index (Wilcoxon rank sum test, *p* < 0.05, Figure 2a). The higher Chao1 index of phytoplanktonic communities was present in both upstream and downstream regions (Figure 2b1). Conversely, the elevated Shannon index of zooplanktonic communities was exclusively observed in the downstream region (Figure 2b2). Within a single planktonic type, a significant higher Shannon index of phytoplankton was found in the upstream region in comparison to the downstream region (Figure 2c2), while the upstream region possessed a higher Chao1 index of zooplankton (Figure 2c3).

Subsequent investigation of variations in the beta-diversity (Bray–Curtis distance) of planktonic microeukaryotes revealed that phytoplankton exhibited higher levels of beta-diversity in comparison to zooplankton throughout the Beipan River (Figure 2d). This phenomenon was also observed in both upstream and downstream regions (Figure 2e). Furthermore, an elevated level of beta-diversity was observed in both phytoplankton and zooplankton in the upstream region in comparison to the downstream region (Figure 2f). In contrast to the higher alpha- and beta-diversity of phytoplankton, a lower gamma-diversity of phytoplankton compared to zooplankton was obtained in the entire Beipan River (Figure 2g) and both upstream and downstream regions (Figure 2h). For a single planktonic type, the gamma-diversity of both phytoplankton and zooplankton was significantly higher in the upstream region compared to that in the downstream region (Figure 2i).

### 3.4 Variations in the composition of planktonic microeukarytoes

**Figure 3:**
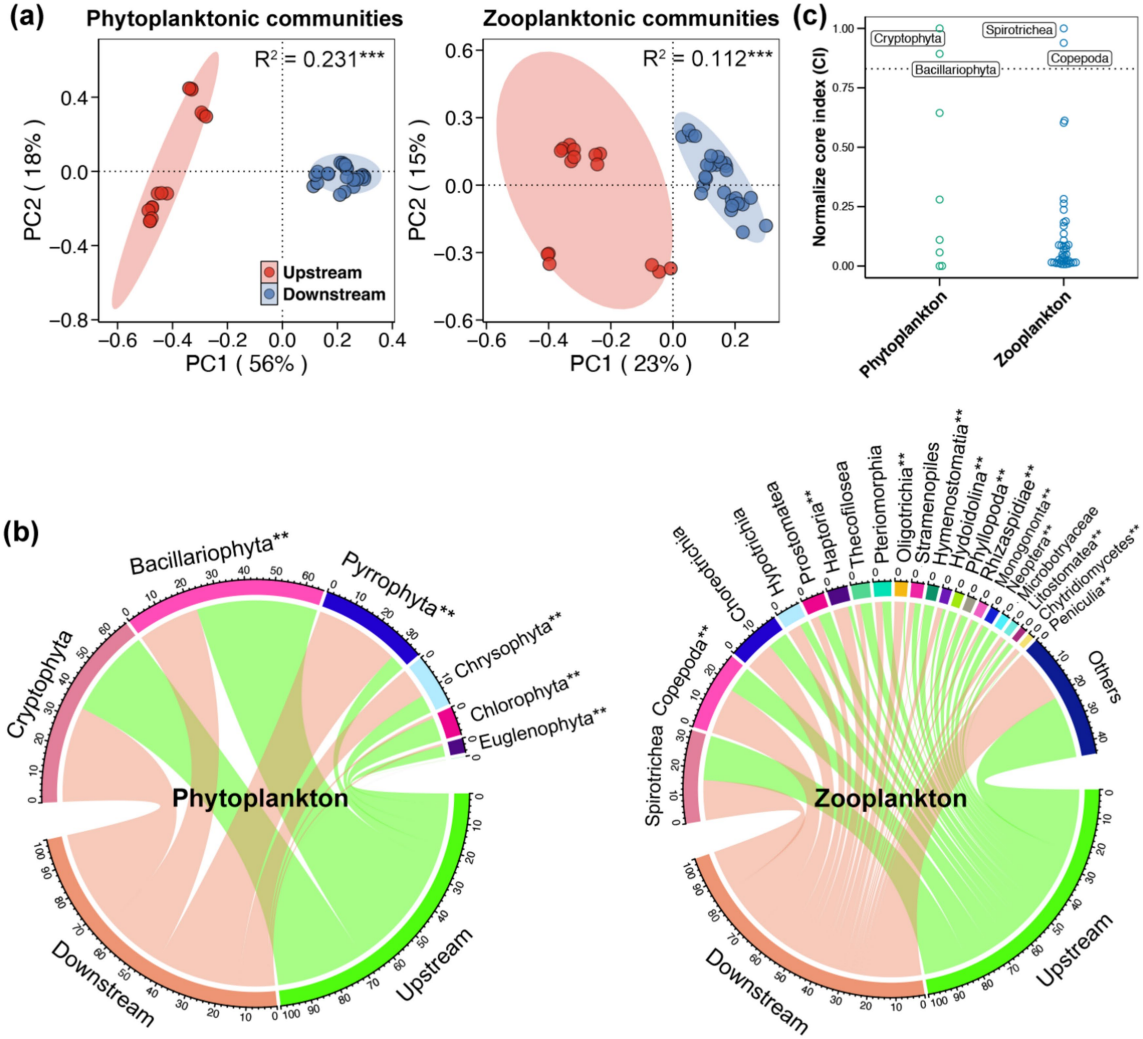
**(a)** Principal coordinates analysis (PCoA) and adonis test based on the Bray-Curtis distance showed the variations in the phytoplanktonic and zooplanktonic communities in the Beipan River. **(b)** Chord diagram showed the composition of the phytoplanktonic and zooplanktonic communities in the Beipan River. ** adjacent to the name of taxa represents significant difference in the relative abundance of corresponding taxa between the upstream and downstream regions (Wilcoxon rank sum test, *p* < 0.05). **(c)** Normalize core index of phytoplankton and zooplankton in the Beipan River.

The PCoA, based on the Bray-Curtis distance, demonstrated distinctly separated clusters of both phytoplanktonic and zooplanktonic communities between the upstream and downstream regions (Figure 3a). The adonis test, based on the Bray-Curtis distance, further confirmed the significant variations in phytoplanktonic and zooplanktonic communities between the upstream and downstream region (*p* < 0.05). The most prevalent phytoplanktonic phylum in the Beipan River was Cryptophyta (33.16%), followed by Bacillariophyta (32.97%), Pyrrophyta (18.02%), and Chrysophyta (8.75%) (Figure 3b). For the classification of zooplankton, Spirotrichea (16.33%) and Copepoda (13.99%) were found to be dominant in the Beipan River, followed by Choreotrichia (9.35%), Hypotrichia (4.25%), and Prostomatea (4.03%) (Figure 3b). The NorCI asserts that the core planktonic taxa in the Beipan River are comprised of Cryptophyta, Bacillariophyta, Spirotrichea and Copepoda (Figure 3c). A total of eight phytoplanktonic phyla were observed, with Bacillariophyta, Chlorophyta and Euglenophyta being more abundant in the upstream region, and Chrysophyta and Pyrrophyta being more abundant in the downstream region (Supplementary Figure S3a). A comparison of the relative abundances of various taxa of copepods revealed that those of Copepoda, Haptoria, Oligotrichia and Hypotrichia were higher in the upstream region, while those of Hymenostomatia, Phyllopoda, Rhizaspididae and Prostomatea were found to be more abundant in the downstream region (Supplementary Figure S3b).

### 3.5 Dispersal-selection relationships on assembly of planktonic microeukarytoes

**Figure 4:**
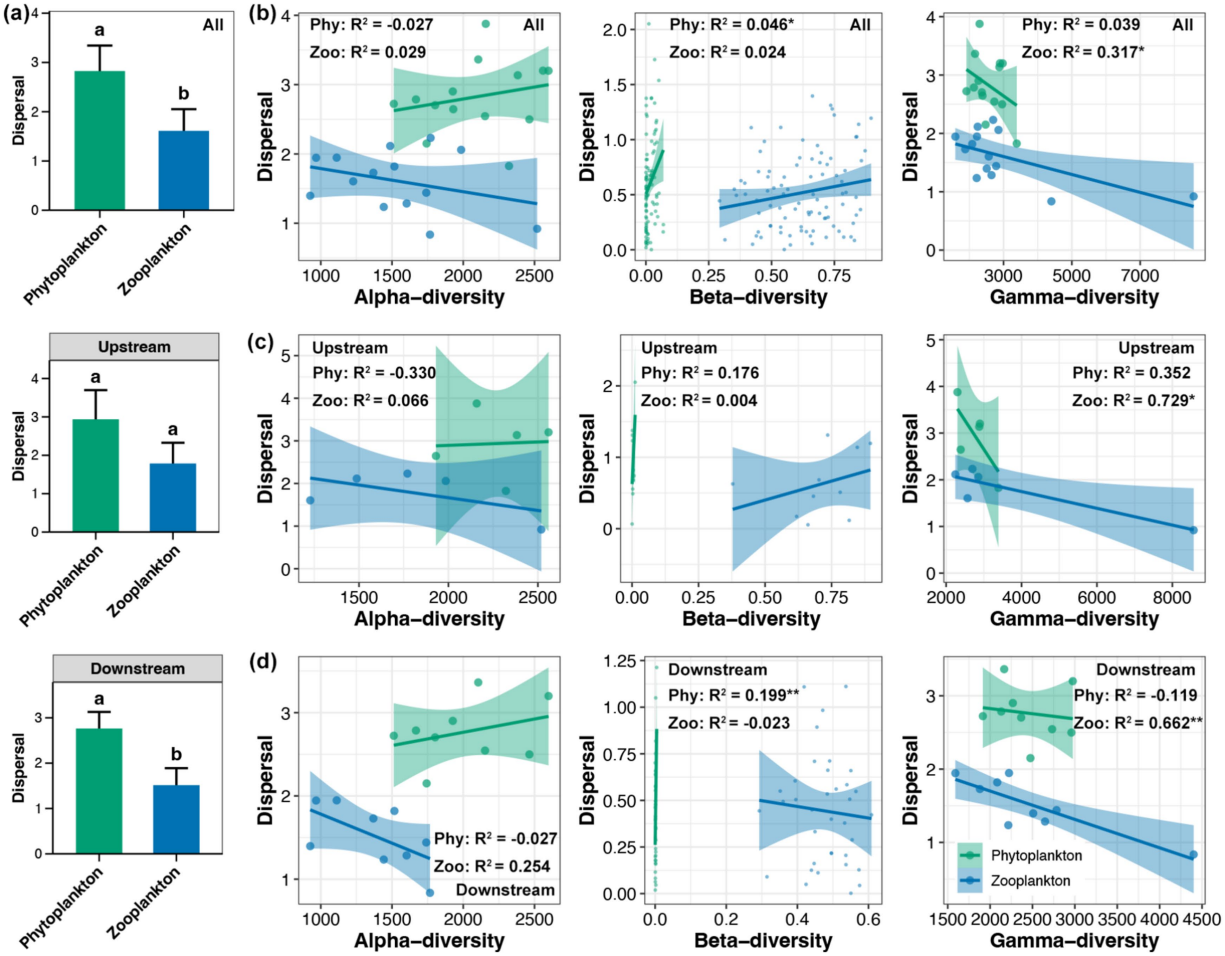
**(a)** Differences in the dispersal between phytoplankton and zooplankton in the whole Beipan River, the upstream region, and the downstream region, respectively. Different lowercase letters in the same subfigure represent significant differences (Wilcoxon rank sum test, *p* < 0.05). Linear regression between the alpha-, beta-, and gamma-diversity of phytoplankton and zooplankton with dispersal in the whole Beipan River **(b)**, the upstream region **(c)**, and the downstream region **(d)**, respectively. * and ** represent the *p*-value lower than 0.05 and 0.01, respectively.

**Figure 5:**
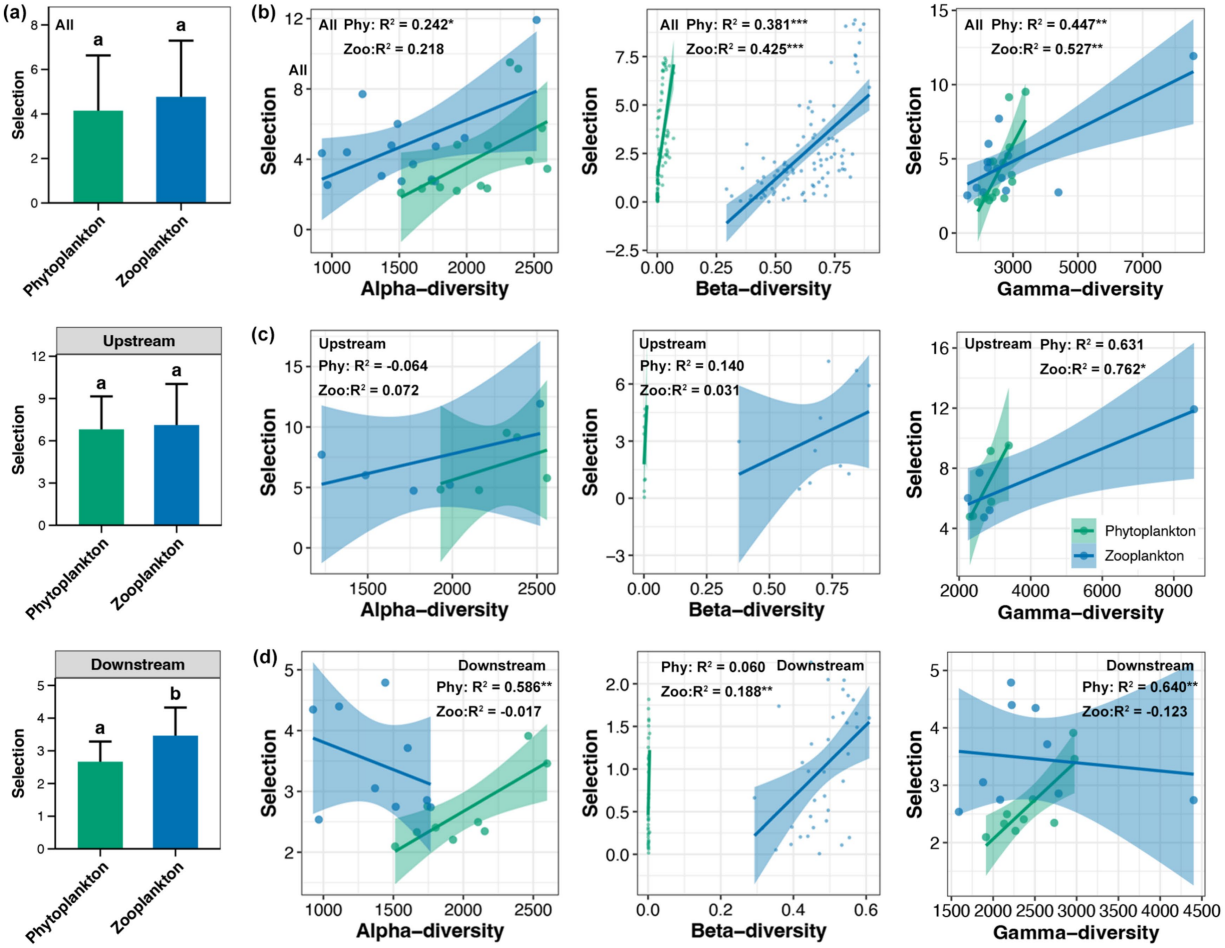
**(a)** Differences in the selection between phytoplankton and zooplankton in the whole Beipan River, the upstream region, and the downstream region, respectively. Different lowercase letters in the same subfigure represent significant differences (Wilcoxon rank sum test, *p* < 0.05). Linear regression between the alpha-, beta-, and gamma-diversity of phytoplankton and zooplankton with selection in the whole Beipan River **(b)**, the upstream region **(c)**, and the downstream region **(d)**, respectively. * and ** represent the *p*-value lower than 0.05 and 0.01, respectively.

The dispersal of phytoplankton was found to be significantly higher than that of zooplankton in the whole Beipan River and the downstream region, but not in the upstream region (Figure 4a). Across the entire length of the Beipan River, a substantial correlation was identified between the dispersal of phytoplankton and their beta-diversity, as well as the dispersal of zooplankton and their gamma-diversity (Figure 4b). In the upstream region, the analysis revealed a significant correlation between gamma-diversity of zooplankton and dispersal (Figure 4c). In the downstream region, results consistent with the whole Beipan River were observed, with a stronger correlation strength (Figure 4d). In contrast to dispersal, a significant difference in the selection of phytoplankton and zooplankton was only observed in the downstream region, but not in the upstream or the entire Beipan River (Figure 5a). With the exception of the alpha-diversity of zooplankton, significant correlations of selection and the alpha-, beta-, and gamma-diversity of phytoplankton and zooplankton were evident in the entire Beipan River (Figure 5b). In the upstream region, the analysis revealed a significant correlation between selection and gamma-diversity of phytoplankton (Figure 5c). In the downstream region, selection was found to be correlated to the alpha- and gamma-diversity of phytoplankton, while beta-diversity of zooplankton (Figure 5d) was also found to be a contributing factor. It has been established that, in general, dispersal processes have a regulatory effect on the beta-diversity of phytoplankton and the gamma-diversity of zooplankton. Conversely, selection processes have been shown to have a regulatory effect on the beta-diversity of zooplankton and the alpha- and gamma-diversity of phytoplankton, particularly in the downstream region of the Beipan River.

### 3.6 Key drivers to regulate the biodiversity of planktonic microeukarytoes

**Figure 6:**
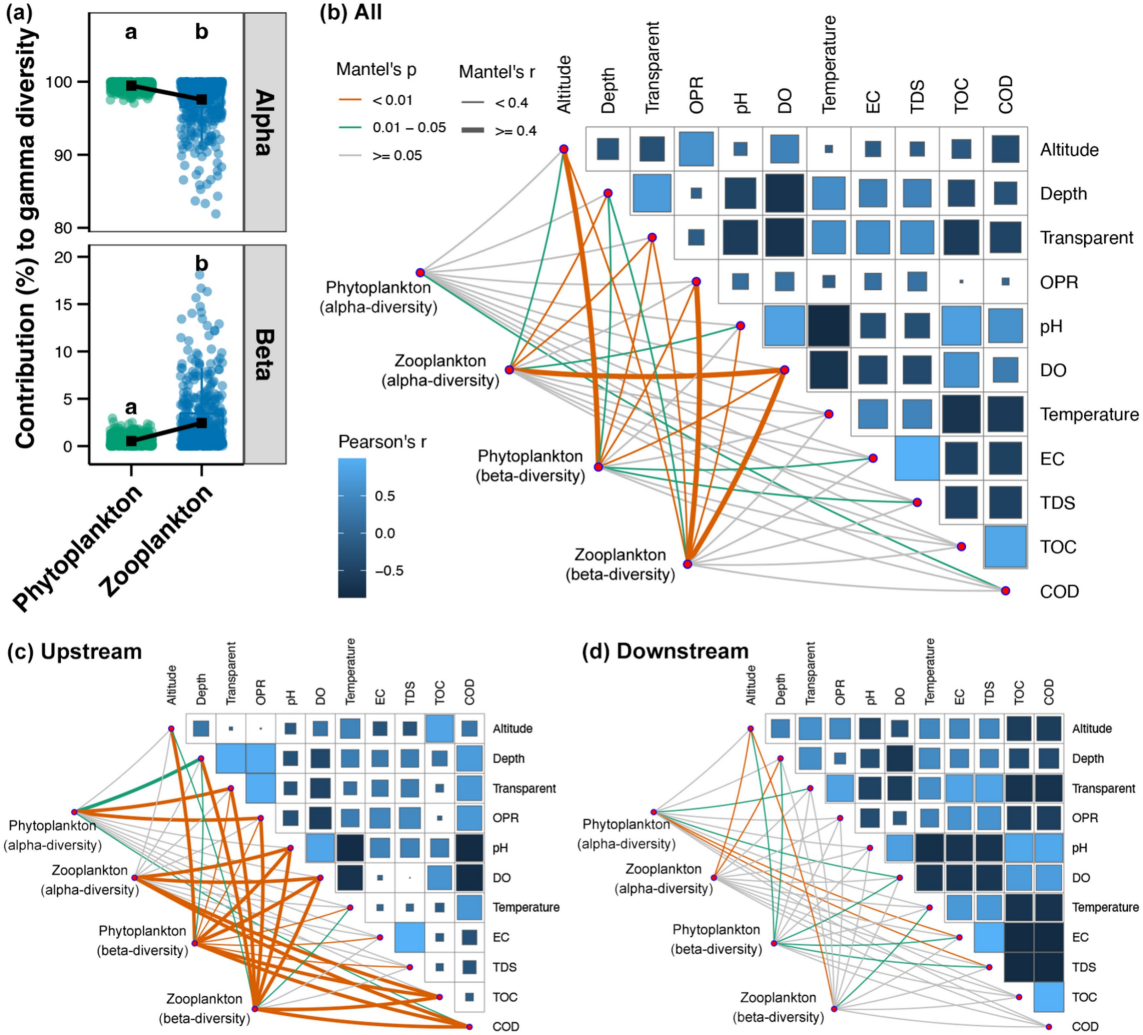
**(a)** Contributions of alpha- and beta-diversity to the gamma-diversity of phytoplankton and zooplankton in the Beipan River. Different lowercase letters in the same subfigure represent significant differences (Wilcoxon rank sum test, *p* < 0.05). Mantel test revealing the correlations between the alpha- and beta-diversity of phytoplankton and zooplankton with environmental variables in the whole Beipan River **(b)**, the upstream region **(c)**, and the downstream region **(d)**, respectively.

As demonstrated in Figure 6a, the alpha-diversity was found to be the primary contributor to the gamma-diversity of both phytoplankton and zooplankton in the Beipan River, as indicated by the decomposition of gamma-diversity. Furthermore, the relative importance of beta-diversity to the gamma-diversity was more significant for zooplankton than phytoplankton (Figure 6a). In the entire Beipan River, COD was found to be significantly correlated to the alpha-diversity of phytoplankton, while the alpha-diversity of zooplankton was significantly correlated to altitude, water depth, transparency, pH and DO (Figure 6b). A significant correlation was identified between altitude, water depth, transparency, OPR, DO, and EC with both phytoplankton and zooplankton beta-diversity (Figure 6b). In the upstream region, the alpha-diversity of phytoplankton exhibited a significant correlation with water depth, transparent, OPR and COD, while the alpha-diversity of zooplankton demonstrated a significant correlation with pH, DO, TOC and COD (Figure 6c). Furthermore, all environmental variables exhibited significant correlations with the beta-diversity of planktonic microeukaryotes, with the exception of EC and TDS, which demonstrated significant correlations with the beta-diversity of zooplankton (Figure 6c). In the downstream region, the alpha- and beta-diversity of phytoplankton were both found to be significantly correlated to DO, EC, and TDS (Figure 6d). Furthermore, the findings revealed a substantial correlation between the altitude and water depth with the beta-diversity of phytoplankton and the alpha-diversity of zooplankton (Figure 6d). Furthermore, the beta-diversity of zooplankton was found to be significantly correlated with altitude and water temperature in the downstream region (Figure 6d). In summary, alterations in environmental conditions exerted an influence on the biodiversity of phytoplankton and zooplankton throughout the Beipan River.

## 4 Discussion

In the Beipan River, the alpha- and beta-diversity of phytoplankton were found to be higher than those of zooplankton (Figures 2a,d). Phytoplankton communities frequently exhibit elevated local species diversity due to their direct response to abiotic factors such as light, nutrients, and hydrological conditions, which exhibit significant variation within river systems (Meng et al., 2020). In contrast, zooplankton are subject to more stringent top-down control from predators and competition, which imposes limitations on their local diversity (Kahsay et al., 2022). In the context of beta-diversity, phytoplankton communities characteristically exhibit elevated spatial turnover, attributable to their superior dispersal capabilities (Shen et al., 2024a). The research undertaken by Du et al. (2023) posits that zooplankton beta diversity is more constrained by biotic homogenization under stronger environmental selection and weaker dispersal. These theories were consistent with the findings of this study, which demonstrated higher dispersal but low selection on phytoplankton compared to zooplankton (Figures 4a, 5a). In contrast to the alpha- and beta-diversity, the gamma-diversity of zooplankton in the Beipan River was significantly higher than that of phytoplankton (Figure 2g). This ostensibly contradictory phenomenon may, in fact, have a basis in reality and be capable of rational explanation. Phytoplankton is frequently constrained by particular environmental conditions, such as light availability (Theus et al., 2022). The light received by an open river is typically not significantly different. The combination of the strong dispersal of phytoplankton results in a comparatively minor disparity between the gamma-diversity at the river scale and the alpha-diversity at a single site. In contrast, zooplankton are found to occupy a wider range of ecological niches, with environmental heterogeneity resulting in the presence of diverse zooplankton species across different sites (McGinty and Irwin, 2025). Consequently, the gamma diversity of zooplankton within the region is significantly higher than the alpha diversity of a single site.

The biodiversity of the phytoplanktonic and zooplanktonic communities at all levels (alpha, beta, and gamma) in the upstream region of the Beipan River has been found to be significantly higher than that in the downstream region (Figure 2). These trends were consistent with the higher environmental heterogeneity observed in the upstream region compared to the downstream region (Figure 1c). It has been demonstrated that environmental heterogeneity can lead to an increase in niche diversification, owing to the creation of a variety of environmental conditions. These conditions are conducive to the coexistence of species that are specialized for different resources or habitats. Consequently, this promotes higher biodiversity at local and regional scales (Wan et al., 2023). However, greater environmental heterogeneity typically requires more species to maintain ecosystem functioning over larger spatial and temporal scales because different species contribute to functioning in different environments (Thompson et al., 2021). It is therefore important to note the existence of a significant trade-off, which is known as the “area-heterogeneity trade-off.” The argument posits that as environmental heterogeneity increases within a given area, the effective habitat size for each species decreases (Allouche et al., 2012). This trade-off signifies that while moderate environmental heterogeneity fosters species richness by providing diverse ecological niches, pronounced environmental heterogeneity within limited geographical areas can curtail biodiversity due to diminished effective population sizes and augmented stochastic extinctions. Anthropogenic disturbances have the capacity to modify the relationship between environmental heterogeneity and biodiversity by altering the range, heterogeneity, and autocorrelation of environmental conditions experienced by species. This, in turn, has the potential to impact biodiversity patterns (Agra et al., 2021). The findings of this study suggested that the environmental heterogeneity in the upstream of the Beipan River was in a healthy state and could maintain a high level of biodiversity. However, the static water environment in the downstream area formed by the partition dam kept the environment in the watershed in a relatively stable state. This phenomenon resulted in a reduction of the habitat niche breadth of the downstream region, leading to a decline in biodiversity of planktonic microeukaryotes.

The biodiversity of phytoplankton and zooplankton in river ecosystems is determined by the interplay between species dispersal capabilities and environmental selection pressures (Borics et al., 2021). Phytoplankton in river systems is found to rely predominantly on passive dispersal via water currents, thereby aligning their community structure closely with hydrological connectivity (Quevedo-Ortiz et al., 2024). In the Beipan River, the entire river system remains fully connected despite the presence of dams, indicating that there are no evident dispersal limitations. Consequently, variations in the biodiversity of phytoplankton between different sites or regions are more influenced by divergent selection pressures resulting from changes in environmental conditions (Ge et al., 2022). The results of this study demonstrated a significant correlation between the biodiversity of phytoplankton and the selection observed, thus confirming the initial hypothesis (Figure 6). Empirical studies have also demonstrated that reduced environmental variability leads to more uniform biodiversity of phytoplankton across diverse river systems (Ding et al., 2021; Yang et al., 2022; Zhao et al., 2017). The beta- and gamma-diversity of zooplankton were also found to be significantly correlated to selection in the Beipan River, but its underlying mechanism was not the same as that of phytoplankton. This phenomenon is frequently elucidated by the species sorting hypothesis, which posits that local abiotic factors exert a pronounced influence on the composition and biodiversity of zooplankton communities (Yang et al., 2018). Moreover, it is important to acknowledge that, despite the discovery of a substantial correlation between the gamma-diversity of zooplankton and dispersal in the downstream region, characterized by low environmental heterogeneity, this correlation was found to be negative (see Figure 4d). On the one hand, the behavior of zooplankton is characterized by active locomotion, enabling them to exploit heterogeneous microhabitats even in macroscale homogeneous rivers (Goździejewska et al., 2024). The aforementioned dynamics serve to emphasize the elevated levels of active dispersal exhibited by zooplankton, thereby signifying the occurrence of species sorting along environmental gradients that are characterized by subtlety (Chaparro et al., 2023). Conversely, the broader niche breadths of zooplanktons serve as a form of buffering against environmental stochasticity. Consequently, environmental uniformity erodes their biodiversity by favoring generalist species with broad fundamental niches (Hagen et al., 2024).

A greater number of correlations were identified between environmental variables and the biodiversity of planktonic microeukaryotes in high-environmental-heterogeneity upstream regions in comparison to environmental stability in downstream areas (Figures 6c,d). It is evident that environmental heterogeneity plays a pivotal role in the promotion of species diversity. This is achieved by the provision of partitioned niche space and spatial variation in environmental conditions. These factors, in turn, support niche differentiation and species sorting (Daleo et al., 2023). Furthermore, increased environmental heterogeneity in rivers has been demonstrated to enhance habitat diversity, thereby supporting greater biodiversity and stronger correlations between environmental variables and species assemblages (Agra et al., 2024; Nunes et al., 2020). Furthermore, a substantial correlation between organic carbon and biodiversity of planktonic microeukaryotes was identified in the upstream region (Figure 6c). It can be posited that the elevated levels of organic carbon, as well as its standard deviation, in the upstream region (Figure 1b), may be a contributing factor to the pronounced environmental heterogeneity observed. Higher organic carbon has been demonstrated to support greater microbial biomass and diversity (Cheng et al., 2024; Shen et al., 2024b). This has been identified as a key environmental driver in rivers, maintaining ecosystem biodiversity and function. The findings of this study indicated that the level of organic carbon in rivers could potentially serve as a crucial regulatory factor for the biodiversity of planktonic microeukaryotes.

## 5 Protection

Core findings based on environmental heterogeneity regulating biodiversity (high heterogeneity upstream → high diversity, low heterogeneity downstream → low diversity). It is recommended to avoid new dams or river channelization in the upstream area to maintain water flow rate changes and habitat diversity. Control organic carbon (TOC) and COD inputs and reduce agricultural and domestic sewage discharge to prevent eutrophication from weakening the heterogeneity effect. Downstream areas enhance habitat complexity and restore riparian zone vegetation to create microhabitat heterogeneity. Implement ecological dispatching (e.g., artificial flood pulses) to simulate natural hydrological fluctuations and break environmental homogenization. Add fishways or ecological channels at dams to promote plankton dispersal. Focus on COD and TOC dynamics throughout the watershed as early warning indicators of water quality. Monitor water depth, transparency, and dissolved oxygen, which significantly influence its alpha diversity. In general, the mechanism of “high environmental heterogeneity → high biodiversity” is maintained in the upstream, and the homogenized habitats in the downstream due to dams are restored, which ultimately enhances the overall ecological resilience of the basin (e.g., buffering capacity against climate change).

## 6 Conclusion

The present study explores variations in the biodiversity of planktonic microeukaryotes along a river exhibiting significant variability, and their associations with the dispersal-selection relationship. The higher dispersal but lower selection on phytoplankton compared to zooplankton resulted in higher alpha- and beta-diversity of phytoplankton than zooplankton throughout the Beipan River. In contrast, it was hypothesized that wider niche breadths of zooplankton combined with environmental heterogeneity contributed to higher gamma-diversity of zooplankton. Furthermore, the relatively stable environmental conditions in the downstream region, induced by the fully barrier-type dam, resulted in a reduction of biodiversity, both in terms of phytoplankton and zooplankton. It is evident that variations in environmental conditions exert a significant influence on the biodiversity of planktonic microeukaryotes. This influence can be attributed to higher levels of environmental heterogeneity, which in turn support more diverse planktonic communities. With the exception of alterations in natural conditions, such as altitude and water depth, the organic carbon level was demonstrated to be a pivotal factor in regulating the biodiversity of planktonic microeukaryotes in highly variable river regions. In summary, the disparity in environmental conditions throughout a river system has been shown to regulate the balance between dispersal and selection, thereby maintaining biodiversity of planktonic microeukaryotes.

## Data Availability

The original contributions presented in the study are publicly available. This data can be found in: https://www.ncbi.nlm.nih.gov/sra accession numbers: SRR33930973–SRR33931014.

## References

[ref1] Agra J. Cornelissen T. Viana-Junior A. B. Callisto M. (2024). A global synthesis and meta-analysis of the environmental heterogeneity effects on the freshwater biodiversity. Oikos 2024:e10186. doi: 10.1111/oik.10186

[ref2] Agra J. Ligeiro R. Heino J. Macedo D. R. Castro D. M. Linares M. S.. (2021). Anthropogenic disturbances alter the relationships between environmental heterogeneity and biodiversity of stream insects. Ecol. Indic. 121:107079. doi: 10.1016/j.ecolind.2020.107079

[ref3] Allouche O. Kalyuzhny M. Moreno-Rueda G. Pizarro M. Kadmon R. (2012). Area–heterogeneity tradeoff and the diversity of ecological communities. Proc. Natl. Acad. Sci. 109, 17495–17500. doi: 10.1073/pnas.1208652109, PMID: 23045670 PMC3491518

[ref4] Amaral-Zettler L. A. McCliment E. A. Ducklow H. W. Huse S. M. (2009). A method for studying protistan diversity using massively parallel sequencing of V9 hypervariable regions of small-subunit ribosomal RNA genes. PLoS One 4:e6372. doi: 10.1371/journal.pone.0006372, PMID: 19633714 PMC2711349

[ref5] Barbour K. M. Martiny J. B. (2024). Investigating eco-evolutionary processes of microbial community assembly in the wild using a model leaf litter system. ISME J. 18:wrae043. doi: 10.1093/ismejo/wrae043, PMID: 38506671 PMC11008689

[ref6] Bokulich N. A. Kaehler B. D. Rideout J. R. Dillon M. Bolyen E. Knight R.. (2018). Optimizing taxonomic classification of marker-gene amplicon sequences with qiime 2’s q2-feature-classifier plugin. Microbiome 6:90. doi: 10.1186/s40168-018-0470-z, PMID: 29773078 PMC5956843

[ref7] Bokulich N. A. Subramanian S. Faith J. J. Gevers D. Gordon J. I. Knight R.. (2013). Quality-filtering vastly improves diversity estimates from Illumina amplicon sequencing. Nat. Methods 10, 57–59. doi: 10.1038/nmeth.2276, PMID: 23202435 PMC3531572

[ref8] Borics G. Abonyi A. Salmaso N. Ptacnik R. (2021). Freshwater phytoplankton diversity: models, drivers and implications for ecosystem properties. Hydrobiologia 848, 53–75. doi: 10.1007/s10750-020-04332-9, PMID: 32836348 PMC7334633

[ref9] Brown J. J. Mihaljevic J. R. Des Marteaux L. Hrček J. (2020). Metacommunity theory for transmission of heritable symbionts within insect communities. Ecol. Evol. 10, 1703–1721. doi: 10.1002/ece3.5754, PMID: 32076545 PMC7029081

[ref10] Cagle G. A. McGrew A. Baiser B. Record S. Gotelli N. J. Gravel D.. (2024). Dispersal limitation governs bacterial community assembly in the northern pitcher plant (*Sarracenia purpurea*) at the continental scale. Glob. Ecol. Biogeogr. 33:e13922. doi: 10.1111/geb.13922

[ref11] Cermeño P. Teixeira I. G. Branco M. Figueiras F. G. Marañón E. (2014). Sampling the limits of species richness in marine phytoplankton communities. J. Plankton Res. 36, 1135–1139. doi: 10.1093/plankt/fbu033

[ref12] Chaparro G. O’Farrell I. Hein T. (2023). Hydrological conditions determine shifts of plankton metacommunity structure in riverine floodplains without affecting patterns of species richness along connectivity gradients. Aquat. Sci. 85:41. doi: 10.1007/s00027-023-00937-z

[ref13] Cheng S. Meng F. Wang Y. Zhang J. Zhang L. (2024). The potential linkage between sediment oxygen demand and microbes and its contribution to the dissolved oxygen depletion in the Gan River. Front. Microbiol. 15:1413447. doi: 10.3389/fmicb.2024.1413447, PMID: 39144217 PMC11322766

[ref14] Daleo P. Alberti J. Chaneton E. J. Iribarne O. Tognetti P. M. Bakker J. D.. (2023). Environmental heterogeneity modulates the effect of plant diversity on the spatial variability of grassland biomass. Nat. Commun. 14:1809. doi: 10.1038/s41467-023-37395-y, PMID: 37002217 PMC10066197

[ref15] Ding Y. Pan B. Zhao G. Sun C. Han X. Li M. (2021). Geo-climatic factors weaken the effectiveness of phytoplankton diversity as a water quality indicator in a large sediment-laden river. Sci. Total Environ. 792:148346. doi: 10.1016/j.scitotenv.2021.148346, PMID: 34144241

[ref16] Dixon P. (2003). VEGAN, a package of R functions for community ecology. J. Veg. Sci. 14, 927–930. doi: 10.1111/j.1654-1103.2003.tb02228.x

[ref17] Doane M. P. Ostrowski M. Brown M. Bramucci A. Bodrossy L. van de Kamp J.. (2023). Defining marine bacterioplankton community assembly rules by contrasting the importance of environmental determinants and biotic interactions. Environ. Microbiol. 25, 1084–1098. doi: 10.1111/1462-2920.16341, PMID: 36700447

[ref18] Dray S. Dufour A. B. (2007). The ade4 package: implementing the duality diagram for ecologists. J. Stat. Softw. 22, 1–20. doi: 10.18637/jss.v022.i04

[ref19] Du C. Zhao F. Shang G. Wang L. Jeppesen E. Zhang L.. (2023). Ammonia influences the zooplankton assemblage and beta diversity patterns in complicated urban river ecosystems. Water 15:1449. doi: 10.3390/w15081449

[ref20] Elzhov T. V. Mullen K. M. Spiess A. Bolker B. (2010). R interface to the Levenberg-Marquardt nonlinear least-squares algorithm found in MINPACK. Plus support for bounds, 1.2-1.

[ref21] Engel F. Attermeyer K. Ayala A. I. Fischer H. Kirchesch V. Pierson D. C.. (2019). Phytoplankton gross primary production increases along cascading impoundments in a temperate, low-discharge river: insights from high frequency water quality monitoring. Sci. Rep. 9:6701. doi: 10.1038/s41598-019-43008-w, PMID: 31040329 PMC6491547

[ref22] Evans S. Martiny J. B. Allison S. D. (2017). Effects of dispersal and selection on stochastic assembly in microbial communities. ISME J. 11, 176–185. doi: 10.1038/ismej.2016.96, PMID: 27494293 PMC5315486

[ref23] Fan Q. Liu K. Wang Z. Liu D. Li T. Hou H.. (2024). Soil microbial subcommunity assembly mechanisms are highly variable and intimately linked to their ecological and functional traits. Mol. Ecol. 33:e17302. doi: 10.1111/mec.17302, PMID: 38421102

[ref24] Fu B. Liu Y. Meadows M. E. (2023). Ecological restoration for sustainable development in China. Natl. Sci. Rev. 10:nwad033. doi: 10.1093/nsr/nwad033, PMID: 37266558 PMC10232043

[ref25] Ge F. Ma Z. Chen B. Wang Y. Lu X. An S.. (2022). Phytoplankton species diversity patterns and associated driving factors in China’s Jiulong River estuary: roles that nutrients and nutrient ratios play. Front. Mar. Sci. 9:829285. doi: 10.3389/fmars.2022.829285

[ref26] Giering S. L. Culverhouse P. F. Johns D. G. McQuatters-Gollop A. Pitois S. G. (2022). Are plankton nets a thing of the past? An assessment of *in situ* imaging of zooplankton for large-scale ecosystem assessment and policy decision-making. Front. Mar. Sci. 9:986206. doi: 10.3389/fmars.2022.986206

[ref27] Goździejewska A. M. Cymes I. Glińska-Lewczuk K. (2024). Zooplankton functional diversity as a bioindicator of freshwater ecosystem health across land use gradient. Sci. Rep. 14:18456. doi: 10.1038/s41598-024-69577-z, PMID: 39117749 PMC11310481

[ref28] Hagen O. Viana D. S. Wiegand T. Chase J. M. Onstein R. E. (2024). The macro-eco-evolutionary interplay between dispersal, competition and landscape structure in generating biodiversity. Philos. Trans. R. Soc. Lond. Ser. B Biol. Sci. 379:20230140. doi: 10.1098/rstb.2023.0140, PMID: 38913052 PMC11391298

[ref29] Huang H. (2021) linkET: Everything is Linkable. R package version 0.0.7.4

[ref30] Huber P. Metz S. Unrein F. Mayora G. Sarmento H. Devercelli M. (2020). Environmental heterogeneity determines the ecological processes that govern bacterial metacommunity assembly in a floodplain river system. ISME J. 14, 2951–2966. doi: 10.1038/s41396-020-0723-2, PMID: 32719401 PMC7784992

[ref31] Kahsay A. Lemmens P. Triest L. De Meester L. Kibret M. Verleyen E.. (2022). Plankton diversity in tropical wetlands under different hydrological conditions (Lake Tana, Ethiopia). Front. Environ. Sci. 10:816892. doi: 10.3389/fenvs.2022.816892

[ref32] Keck F. Millet L. Debroas D. Etienne D. Galop D. Rius D.. (2020). Assessing the response of micro-eukaryotic diversity to the great acceleration using lake sedimentary DNA. Nat. Commun. 11:3831. doi: 10.1038/s41467-020-17682-8, PMID: 32737305 PMC7395174

[ref33] Lin Y. J. Chen T. C. Chen C. T. A. Wong S. L. Meng P. J. Chen M. H. (2024). Long-term monitoring dataset of plankton assemblages in western Taiwan coastal water. Scientific Data 11:917. doi: 10.1038/s41597-024-03784-1, PMID: 39179583 PMC11344027

[ref34] Luan L. Jiang Y. Cheng M. Dini-Andreote F. Sui Y. Xu Q.. (2020). Organism body size structures the soil microbial and nematode community assembly at a continental and global scale. Nat. Commun. 11:6406. doi: 10.1038/s41467-020-20271-4, PMID: 33335105 PMC7747634

[ref35] Madhav S. Kanhaiya S. Srivastav A. L. Singh V. B. Singh P. (2022). Ecological significance of river ecosystems: challenges and management strategies. Amsterdam: Elsevier.

[ref36] McGinty N. Irwin A. (2025). Global variation in zooplankton niche divergence across ocean basins. Ecol. Lett. 28:e70089. doi: 10.1111/ele.70089, PMID: 39976335 PMC11841027

[ref37] Menéndez-Serra M. Ontiveros V. J. Cáliz J. Alonso D. Casamayor E. O. (2023). Understanding stochastic and deterministic assembly processes in microbial communities along temporal, spatial and environmental scales. Mol. Ecol. 32, 1629–1638. doi: 10.1111/mec.16842, PMID: 36626114

[ref38] Meng F. Li Z. Li L. Lu F. Liu Y. Lu X.. (2020). Phytoplankton alpha diversity indices response the trophic state variation in hydrologically connected aquatic habitats in the Harbin section of the Songhua River. Sci. Rep. 10:21337. doi: 10.1038/s41598-020-78300-7, PMID: 33288790 PMC7721905

[ref39] Naka L. N. Werneck F. P. Rosser N. Pil M. W. Boubli J. P. (2022). The role of rivers in the origins, evolution, adaptation, and distribution of biodiversity. Front. Ecol. Evol. 10:1035859.

[ref40] Nunes L. S. C. Umetsu C. A. Camargo A. F. M. (2020). Environmental heterogeneity influences life-form richness and species composition but not species richness of aquatic macrophytes in tropical coastal rivers. Freshw. Biol. 65, 1894–1905. doi: 10.1111/fwb.13586

[ref41] Oldenburg E. Kronberg R. M. Metfies K. Wietz M. von Appen W. J. Bienhold C.. (2024). Beyond blooms: the winter ecosystem reset determines microeukaryotic community dynamics in the Fram Strait. Commun. Earth Environ. 5:643. doi: 10.1038/s43247-024-01782-0

[ref42] Paradis E. Schliep K. (2019). Ape 5.0: an environment for modern phylogenetics and evolutionary analyses in R. Bioinformatics 35, 526–528. doi: 10.1093/bioinformatics/bty633, PMID: 30016406

[ref43] Peipoch M. Ensign S. H. (2022). Deciphering the origin of riverine phytoplankton using in situ chlorophyll sensors. Limnol. Oceanogr. Lett. 7, 159–166. doi: 10.1002/lol2.10240

[ref44] Quevedo-Ortiz G. Fernández-Calero J. M. Cañedo-Argüelles M. von Schiller D. Fortuño P. Bonada N.. (2024). An experimental study to assess resistance and resilience strategies of freshwater diatoms to cope with drying in Mediterranean temporary rivers. Hydrobiologia 851, 4293–4306. doi: 10.1007/s10750-024-05585-4

[ref45] R Core Team (2022). R: A language and environment for statistical computing. R Foundation for Statistical Computing, Vienna, Austria. Available online at: https://www.R-project.org/ (Accessed May, 2025).

[ref46] Ron R. Fragman-Sapir O. Kadmon R. (2018). Dispersal increases ecological selection by increasing effective community size. Proc. Natl. Acad. Sci. 115, 11280–11285. doi: 10.1073/pnas.1812511115, PMID: 30322907 PMC6217402

[ref47] Shen Y. Zhang Y. Zhou X. Li Q. Zhang J. Cheng R.. (2024a). Environmental DNA metabarcoding revealing the distinct responses of phytoplankton and zooplankton to cascade dams along a river-way. Ecol. Indic. 166:112545. doi: 10.1016/j.ecolind.2024.112545

[ref48] Shen Y. Zhou X. Zhang J. Li Q. Zhang Y. Zuo Q. (2024b). Insights into the effects of environmental factors on phytoplankton and microzooplankton at a basin scale: diversity, assembly mechanisms, and co-occurrence networks. Front. Mar. Sci. 11:1462432. doi: 10.3389/fmars.2024.1462432

[ref49] Sloan W. T. Lunn M. Woodcock S. Head I. M. Nee S. Curtis T. P. (2006). Quantifying the roles of immigration and chance in shaping prokaryote community structure. Environ. Microbiol. 8, 732–740. doi: 10.1111/j.1462-2920.2005.00956.x, PMID: 16584484

[ref50] Sun Y. Li H. Wang X. Jin Y. Nagai S. Lin S. (2023). Phytoplankton and microzooplankton community structure and assembly mechanisms in northwestern Pacific Ocean estuaries with environmental heterogeneity and geographic segregation. Microbiol. Spectr. 11, e04926–e04922. doi: 10.1128/spectrum.04926-22, PMID: 36939346 PMC10100884

[ref51] Theus M. E. Layden T. J. McWilliams N. Crafton-Tempel S. Kremer C. T. Fey S. B. (2022). Photoperiod influences the shape and scaling of freshwater phytoplankton responses to light and temperature. Oikos 2022:e08839. doi: 10.1111/oik.08839

[ref52] Thompson P. L. Kéfi S. Zelnik Y. R. Dee L. E. Wang S. De Mazancourt C.. (2021). Scaling up biodiversity–ecosystem functioning relationships: the role of environmental heterogeneity in space and time. Proc. R. Soc. Lond. B Biol. Sci. 288:20202779. doi: 10.1098/rspb.2020.2779, PMID: 33715425 PMC7944106

[ref53] Vellend M. (2010). Conceptual synthesis in community ecology. Q. Rev. Biol. 85, 183–206. doi: 10.1086/652373, PMID: 20565040

[ref54] Wan J. Z. Wang C. J. Marquet P. A. (2023). Environmental heterogeneity as a driver of terrestrial biodiversity on a global scale. Prog. Phys. Geogr. 47, 912–930. doi: 10.1177/03091333231189045

[ref55] Wang S. Gu S. Zhang Y. Deng Y. Qiu W. Sun Q.. (2024). Microeukaryotic plankton community dynamics under ecological water replenishment: insights from eDNA metabarcoding. Environ. Sci. Ecotechnol. 20:100409. doi: 10.1016/j.ese.2024.100409, PMID: 38572085 PMC10987827

[ref56] Wang J. Pan Z. Yu J. Zhang Z. Li Y. Z. (2023). Global assembly of microbial communities. Msystems 8, e01289–e01222. 37195192 10.1128/msystems.01289-22 PMC10308889

[ref57] Wickham H. (2016). ggplot2: Elegant graphics for data analysis. New York: Springer–Verlag.

[ref58] Xiong J. Li X. Yan M. Lu J. Qiu Q. Chen J. (2020). Comparable ecological processes govern the temporal succession of gut bacteria and micromicroeukarytoes as shrimp aged. Microb. Ecol. 80, 935–945. 32494840 10.1007/s00248-020-01533-6

[ref59] Xu H. Zhang S. Ma G. Zhang Y. Li Y. Pei H. (2020). 18S rRNA gene sequencing reveals significant influence of anthropogenic effects on microeukaryote diversity and composition along a river-to-estuary gradient ecosystem. Sci. Total Environ. 705:135910. doi: 10.1016/j.scitotenv.2019.135910, PMID: 31837544

[ref60] Yang Y. Chen H. Al M. A. Ndayishimiye J. C. Yang J. R. Isabwe A.. (2022). Urbanization reduces resource use efficiency of phytoplankton community by altering the environment and decreasing biodiversity. J. Environ. Sci. 112, 140–151. doi: 10.1016/j.jes.2021.05.001, PMID: 34955197

[ref61] Yang Y. Ni P. Gao Y. Xiong W. Zhao Y. Zhan A. (2018). Geographical distribution of zooplankton biodiversity in highly polluted running water ecosystems: validation of fine-scale species sorting hypothesis. Ecol. Evol. 8, 4830–4840. doi: 10.1002/ece3.4037, PMID: 29876061 PMC5980572

[ref62] Yilmaz P. Parfrey L. W. Yarza P. Gerken J. Pruesse E. Quast C.. (2014). The SILVA and “all-species living tree project (LTOP).” taxonomic frameworks. Nucleic Acids Res. 42, D643–D648. 24293649 10.1093/nar/gkt1209 PMC3965112

[ref63] Yue Z. Fang Q. Zhang S. Wu C. Wang L. (2025). A novel approach to integrating the stability of river ecosystem and its driving factors. Front. Environ. Sci. 13:1524086. doi: 10.3389/fenvs.2025.1524086

[ref64] Zhang Y. Qu Z. Li J. Hu T. Chen C. Lin X. (2023). From river to ocean: connectivity and heterogeneity of aquatic ecosystems depicted by planktonic micromicroeukarytoes. Ecol. Indic. 148:110136.

[ref65] Zhang G. Wei G. Wei F. Chen Z. He M. Jiao S.. (2021). Dispersal limitation plays stronger role in the community assembly of fungi relative to bacteria in rhizosphere across the arable area of medicinal plant. Front. Microbiol. 12:713523. doi: 10.3389/fmicb.2021.713523, PMID: 34484152 PMC8415459

[ref66] Zhang Q. Zhang Z. Lu T. Yu Y. Penuelas J. Zhu Y. G.. (2021). Gammaproteobacteria, a core taxon in the guts of soil fauna, are potential responders to environmental concentrations of soil pollutants. Microbiome 9, 1–17. doi: 10.1186/s40168-021-01150-6, PMID: 34593032 PMC8485531

[ref67] Zhao W. Li Y. Jiao Y. Zhou B. Vogt R. D. Liu H.. (2017). Spatial and temporal variations in environmental variables in relation to phytoplankton community structure in a eutrophic river-type reservoir. Water 9:754. doi: 10.3390/w9100754

[ref68] Zhao Z. Li H. Sun Y. Shao K. Wang X. Ma X.. (2022). How habitat heterogeneity shapes bacterial and protistan communities in temperate coastal areas near estuaries. Environ. Microbiol. 24, 1775–1789. doi: 10.1111/1462-2920.15892, PMID: 34996132

[ref69] Zou S. Lian Q. Ni M. Zhou D. Liu M. Zhang X.. (2024). Spatiotemporal assembly and functional composition of planktonic microeukaryotic communities along productivity gradients in a subtropical lake. Front. Microbiol. 15:1351772. doi: 10.3389/fmicb.2024.1351772, PMID: 38440145 PMC10909917

