# Supplementary material for: Changes in environmental conditions regulate the biodiversity of planktonic microeukaryotes mediated by the dispersal-selection relationships in river: an example of the Beipan River, Guizhou, China
Source: Front Microbiol. 2025 Aug 19;16:1649800. doi: 10.3389/fmicb.2025.1649800 (PMC12402667; doi:10.3389/fmicb.2025.1649800)
Supplement: Supplementary file 1 [file Supplementary_file_1.docx]

#### **Supporting information for**

#### **Changes in environmental conditions regulate the biodiversity of planktonic microeukarytoes mediated by the dispersal-selection relationships in river: An example of the Beipanjiang River, Guizhou, China**

Xiaohan Dong^1,2^, Jiaxin Huang^1,2^, Xinxin Zhou^1,2^, Jiali Ran^1,2^, Ziwei Wang^1,2^, Zongqiang Qi^1,2^, Yanjun Shen^1,2,3^*

^1^Laboratory of Water Ecological Health and Environmental Safety, School of Life Sciences, Chongqing Normal University, Chongqing, 401331, China.

^2^Chongqing Key Laboratory of Conservation and Utilization of Freshwater Fishes, Chongqing, 401331, China.

^3^Animal Biology Key Laboratory of Chongqing Education Commission, Chongqing Normal University, Chongqing, 401331, China

*Corresponding author, Laboratory of Water Ecological Health and Environmental Safety, School of Life Sciences, Chongqing Normal University, Chongqing, 401331, China E-mail address: shenyanjun@cqnu.edu.cn(Yanjun Shen).

**Table S1.** **Environmental factors at each sampling point**.

|  | **Altitude（m）** | **Depth（m）** | **Transparent（m）** | **Salinity**  **(%)** | **OPR**  **(mV)** | **PH** | **DO**  **(mg/L)** | **Temp**  **(℃)** | **EC**  **(us/cm)** | **TDS（ppm）** | **TOC**  **(mg/L)** | **COD**  **(mg/L)** |
| --- | --- | --- | --- | --- | --- | --- | --- | --- | --- | --- | --- | --- |
| S14 | 874 | 2 | 0.2 | 0.01 | 366 | 8.56 | 6.9 | 18.3 | 390 | 195 | 2.6 | 1.9 |
| S13 | 905.6 | 2 | 0.2 | 0.01 | 240 | 8.8 | 9.8 | 16.3 | 321 | 160 | 5.7 | 1 |
| S12 | 871.4 | 0.5 | 0.3 | 0.01 | 243 | 8.53 | 7.3 | 17.1 | 30 | 15 | 3.1 | 2.3 |
| S11 | 881.6 | 25 | 2 | 0.02 | 796 | 8.59 | 6.8 | 17.6 | 377 | 198 | 4 | 2.9 |
| S10 | 786.1 | 0.6 | 0.6 | 0.02 | 386 | 8.73 | 7.8 | 16 | 395 | 198 | 1.6 | 1.2 |
| S9 | 774.6 | 50 | 4 | 0.02 | 188 | 8.54 | 5.5 | 18.1 | 374 | 187 | 1.4 | 1 |
| S8 | 534.4 | 5 | 4 | 0.02 | 258 | 8.56 | 7.1 | 17.3 | 395 | 198 | 1.7 | 1.3 |
| S7 | 550.5 | 3 | 2 | 0.02 | 154 | 8.42 | 6.2 | 17.6 | 407 | 204 | 1.3 | 1 |
| S6 | 546.3 | 0.8 | 0.8 | 0.01 | 184 | 8.64 | 7 | 19.5 | 365 | 183 | 0.96 | 0.72 |
| S5 | 473.4 | 4.5 | 2.5 | 0.02 | 170 | 8.46 | 6 | 19.7 | 389 | 195 | 1 | 0.8 |
| S4 | 534.8 | 25 | 3 | 0.01 | 182 | 8.34 | 5 | 21 | 381 | 191 | 1.2 | 0.9 |
| S3 | 560.4 | 30 | 2 | 0.01 | 186 | 8.37 | 6.1 | 20.1 | 363 | 182 | 1.2 | 0.9 |
| S2 | 342.5 | 32 | 3.5 | 0.02 | 183 | 8.41 | 5.3 | 19.1 | 424 | 212 | 1.2 | 0.9 |
| S1 | 336.2 | 35 | 3.5 | 0.02 | 181 | 8.51 | 4.9 | 19 | 440 | 220 | 1.2 | 0.9 |

**Table S2.** Statistics of effective sequences for phytoplankton and zooplankton.

| **SampleID** | **Phytoplankton** | **Zooplankton** |
| --- | --- | --- |
| S14-1 | 26,311 | 16,159 |
| S14-2 | 25,407 | 15,566 |
| S14-3 | 23,099 | 14,642 |
| S13-1 | 16,451 | 19,678 |
| S13-2 | 19,862 | 22,491 |
| S13-3 | 14,840 | 25,287 |
| S12-1 | 32,123 | 9,592 |
| S12-2 | 35,822 | 11,430 |
| S12-3 | 25,143 | 9,705 |
| S11-1 | 23,538 | 15,585 |
| S11-2 | 28,563 | 14,982 |
| S11-3 | 24,747 | 13,500 |
| S10-1 | 21,307 | 24,425 |
| S10-2 | 20,619 | 22,633 |
| S10-3 | 18,405 | 27,167 |
| S9-1 | 27,032 | 10,280 |
| S9-2 | 23,232 | 10,791 |
| S9-3 | 23,594 | 10,931 |
| S8-1 | 22,225 | 12,711 |
| S8-2 | 25,835 | 12,865 |
| S8-3 | 24,681 | 12,655 |
| S7-1 | 26,136 | 17,682 |
| S7-2 | 29,523 | 12,692 |
| S7-3 | 24,110 | 12,018 |
| S6-1 | 22,041 | 14,611 |
| S6-2 | 18,316 | 17,499 |
| S6-3 | 19,919 | 16,493 |
| S5-1 | 23,939 | 22,588 |
| S5-2 | 23,195 | 16,491 |
| S5-3 | 23,326 | 12,649 |
| S4-1 | 21,127 | 17,817 |
| S4-2 | 18,467 | 12,985 |
| S4-3 | 17,435 | 19,093 |
| S3-1 | 18,615 | 12,636 |
| S3-2 | 24,538 | 12,125 |
| S3-3 | 22,417 | 14,548 |
| S2-1 | 29,781 | 14,044 |
| S2-2 | 33,133 | 12,915 |
| S2-3 | 27,795 | 12,901 |
| S1-1 | 26,362 | 13,735 |
| S1-2 | 28,240 | 15,006 |
| S1-3 | 24,651 | 13,419 |


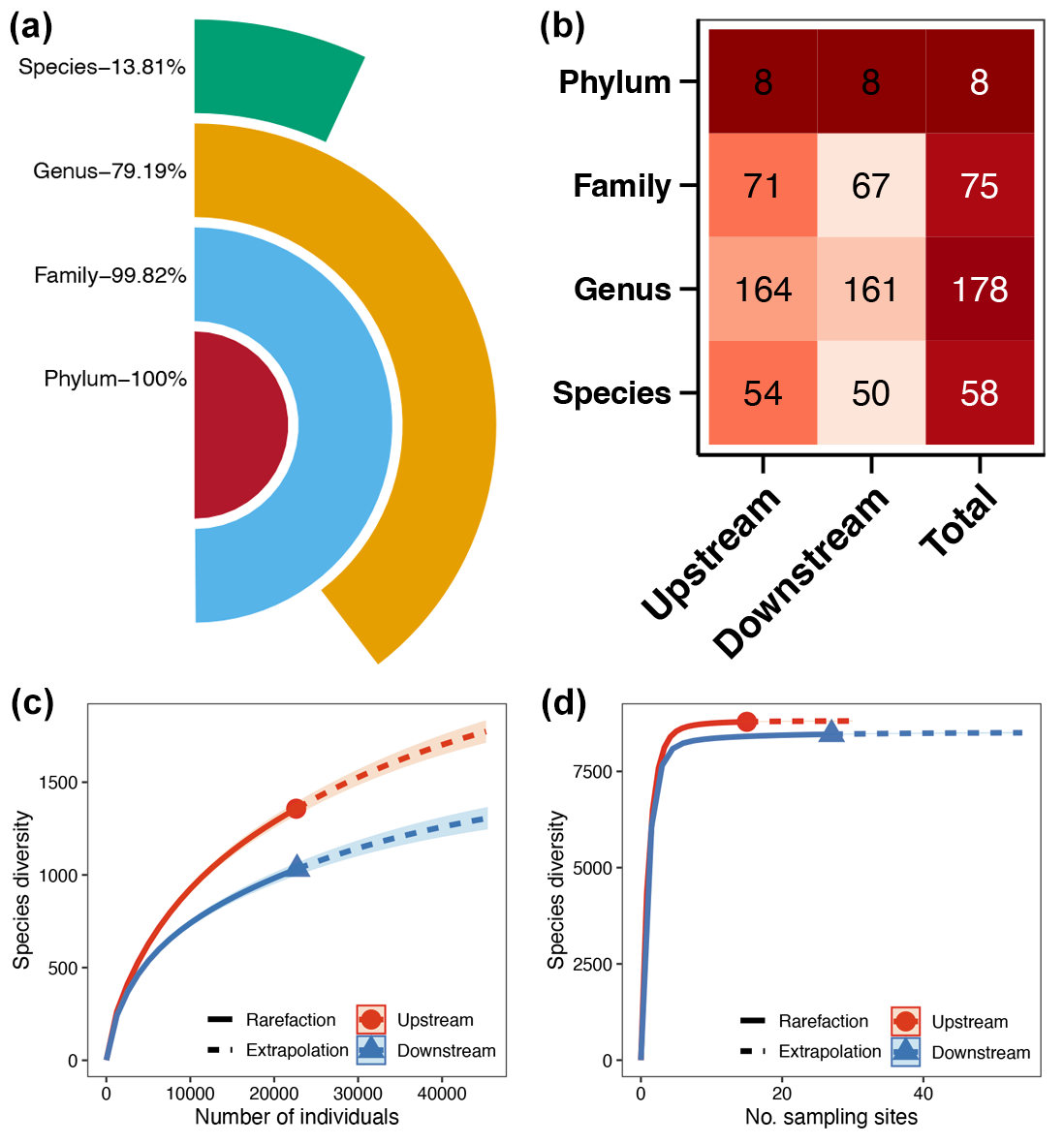


**Figure S1.** (a) Annotation ratio of phytoplanktonic ASVs at different taxonomic levels. (b) Heatmap reveals the detected number of phytoplankton at different taxonomic levels. (c) Rarefaction curve for phytoplankton. (d) Species accumulation curve for phytoplankton.


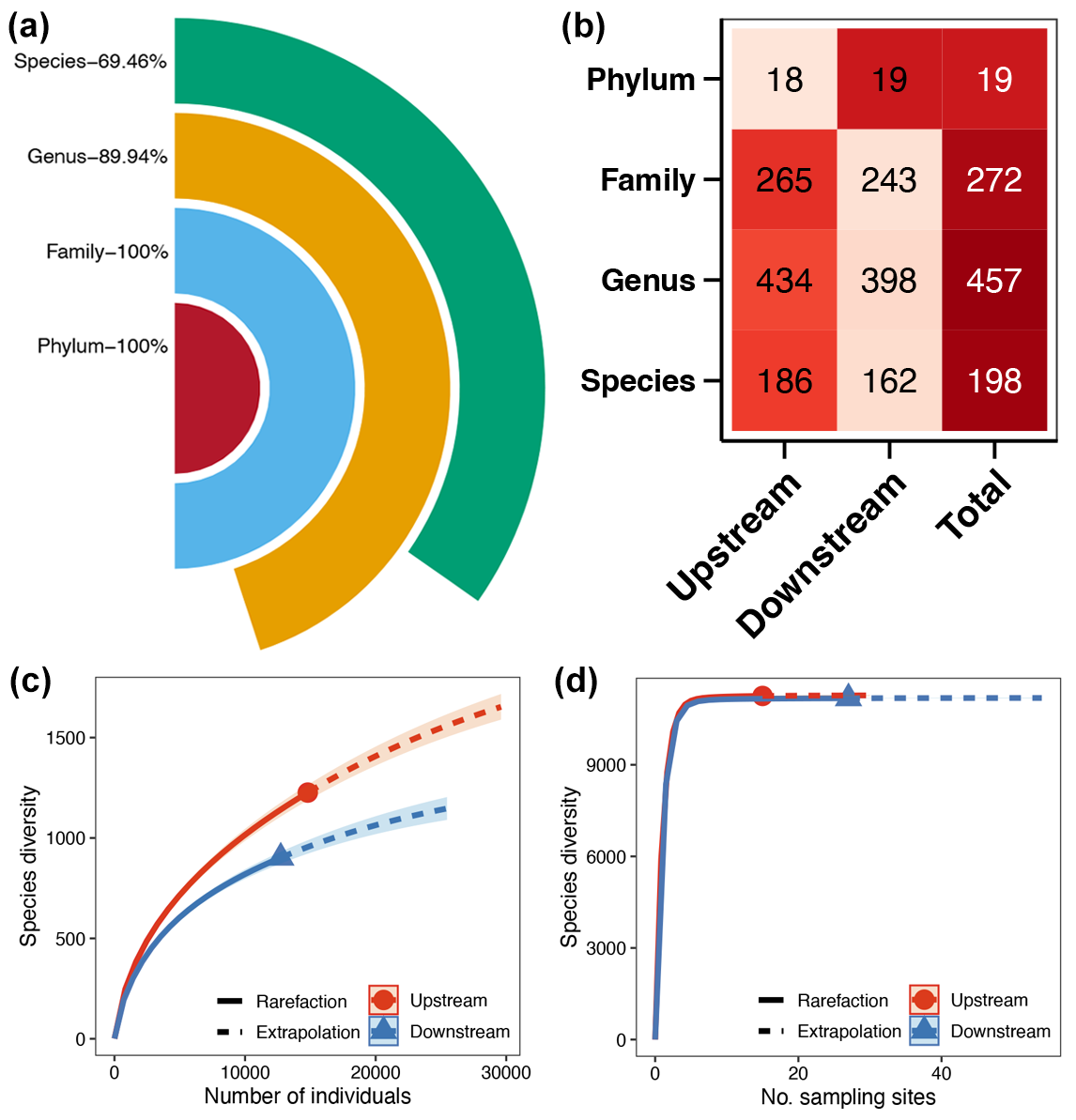


**Figure S2.** (a) Annotation ratio of zooplanktonic ASVs at different taxonomic levels. (b) Heatmap reveals the detected number of zooplankton at different taxonomic levels. (c) Rarefaction curve for zooplankton. (d) Species accumulation curve for zooplankton.


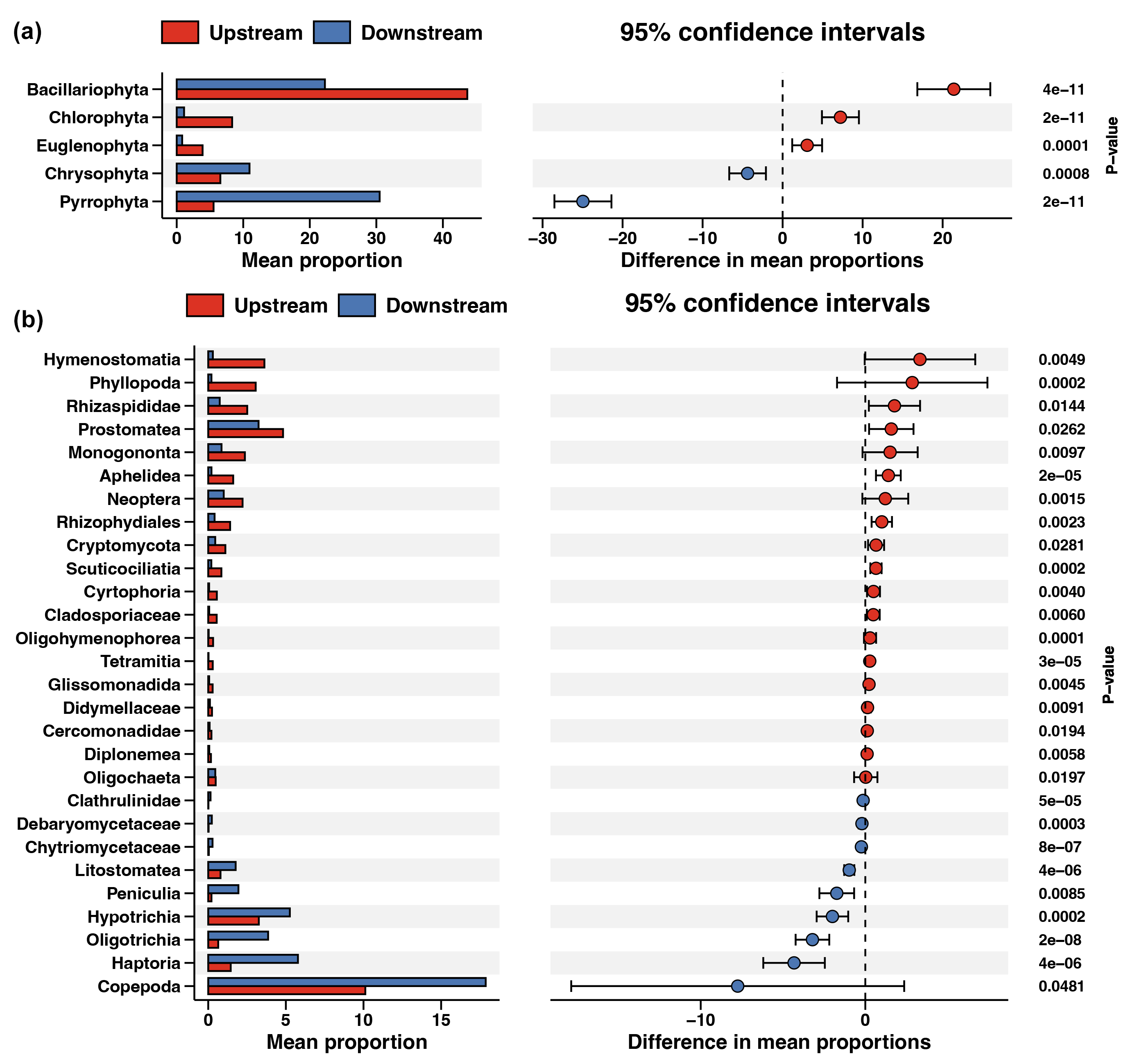


**Figure S3.** Differences in the relative abundance of main phytoplanktonic (a) and zooplanktonic (b) lineages between upstream and downstream regions based on the Wilcoxon rank sum test.
